# Application of Vieth staging in forensic age estimation in the living using MRI of the distal radial epiphysis

**DOI:** 10.1007/s00414-024-03342-9

**Published:** 2024-10-05

**Authors:** Oguzhan Ekizoglu, Ali Er, Mustafa Bozdag, Silke Grabherr

**Affiliations:** 1 Department of Forensic Medicine, Tepecik Training and Research Hospital, Izmir, Turkey; 2 University Centre of Legal Medicine Lausanne-Geneva, Geneva University Hospital and University of Geneva, Geneva, Switzerland; 3 Department of Radiology, Tepecik Training and Research Hospital, Izmir, Turkey

**Keywords:** Forensic age estimation, MRI, Distal radial epiphysis, Vieth staging system

## Abstract

Forensic age estimation is crucial in various legal and civil contexts, particularly in regions experiencing significant migration and inadequate birth registration systems. This study evaluates the applicability of the Vieth staging system for forensic age estimation in the living using MRI of the distal radial epiphysis. A retrospective analysis was conducted on 620 left wrist MRI scans from individuals aged 9.92 to 29.58 years. The study demonstrated high intra- and inter-observer agreement values (κ = 0.974 and κ = 0.961), confirming the method’s reliability. Spearman’s rank correlation analysis showed significant positive correlations between age and ossification stage for both sexes. The minimum ages observed for males were 9.92 years at stage 2, 15.00 years at stage 3, 15.00 years at stage 4, 17.00 years at stage 5, and 20.00 years at stage 6. For females, the minimum ages were 10.08 years at stage 2, 12.33 years at stage 3, 14.25 years at stage 4, 16.33 years at stage 5, and 18.42 years at stage 6. The study supports the applicability of the Vieth methodology for forensic age estimation in the living and suggests that MRI could be a non-invasive and potentially effective tool for determining critical age thresholds in forensic contexts. Further research is recommended to refine these methods and explore their applicability across different populations.

## Introduction

Forensic age estimation is a crucial component in various legal and civil contexts, particularly in countries experiencing significant migration and those with inadequate birth registration systems. Accurate age determination is essential for establishing the legal status of individuals, especially minors, to ensure appropriate legal treatment and access to services. This process becomes increasingly important in the context of refugee movements, where unaccompanied minors require protection and proper legal representation [1, 2].

The Study Group on Forensic Age Diagnostics of the German Societyof Legal Medicine (AGFAD) recommends a comprehensive approach combining physical examination, radiographic examination of the left hand, dental examination, and orthopantomography. If hand ossification is complete, radiographic examination of the clavicle using conventional radiography or computed tomography (CT) is advised [3]. However, the use of ionizing radiation in age estimation has raised ethical concerns, prompting the exploration of radiation-free methods such as magnetic resonance imaging (MRI) [4].

MRI has emerged as a promising tool for forensic age estimation, offering the advantage of being a non-invasive, radiation-free method. Various studies have explored the use of MRI for assessing the ossification of different epiphyseal regions, including the knee, ankle, clavicle, and iliac crest [5–10].

MRI studies examining the development of the distal radius through various methods for forensic age estimation and age assesments in athletes have yielded valuable results [11–18]. The Vieth staging system identifies specific stages of ossification that correlate with age, facilitating the determination of critical age thresholds [10]. For example, the presence of triple banding in the distal radial epiphysis, consisting of hypointense, hyperintense, and hypointense bands, has been associated with specific age ranges. This method has demonstrated high inter- and intra-observer reliability, making it a valuable tool for forensic age estimation [10, 19].

In this study, we aimed to re-evaluate the applicability of the Vieth staging system for forensic age estimation in the living using MRI of the distal radial epiphysis, similar to the approach used by Ottow et al. (2022). By analyzing wrist MRI scans from a diverse population, we sought to assess the accuracy and reliability of this method in determining age thresholds crucial for legal and civil proceedings. Although the methodology has been previously applied, the present study further investigates its utility in a different population. These research questions are important because our findings may contribute to the growing body of knowledge on MRI-based age estimation and support the development of standardized, non-invasive, and radiation-free protocols for forensic applications.

## Materials and methods

### Subjects

This retrospective study examined 688 left wrist MRI scans from individuals aged 9 to 30 years, all with verified birth dates. Ethical approval was obtained from the local ethics committee, and the study adhered to the guidelines set by the institutional research committee and the 1964 Declaration of Helsinki, including its later amendments. Formal consent was not required for this type of study.

MRIs were conducted on trauma patients from 2018 to 2022 at the Tepecik Training and Research Hospital Radiology Clinic. Exclusion criteria included wrist pathologies, motion artifacts in the MRI, systemic or neoplastic disorders affecting bone health (such as endocrine diseases, arthritis, or leukemia), and patients undergoing steroid therapy, radiotherapy, or chemotherapy. The study excluded 68 patients due to bone marrow edema (30), surgical fixation (5), hypothyroidism (5), or motion artifacts in MRI (28). Socioeconomic status and ethnicity data were not available. The final analysis included 620 patients (286 males and 334 females; age range: 9.92 to 29.58 years). All cases admitted to the hospital system using national identification numbers have verified birth date information through the national population registry system. The hospital’s information system calculated chronological ages based on the birth date and MRI examination date.

### Data acquisition

MRIs of the left wrist were performed using a Siemens MAGNETOM Aera 1.5 T machine (Siemens Medical Systems, Erlangen, Germany) with an extremity coil (4-channel flex coil; Siemens). Fat-saturated FSE PD MR images were obtained in the coronal plane with the following parameters: TR, 2400 ms; TE, 39 ms; FoV, 140 × 100 mm; voxel size, 0.5 × 0.5 × 3.0 mm³; section thickness, 1.5 mm; intersection gap, 10 mm; matrix, 320; number of signal averages, 2; acquisition time, 2.38 s; and reading direction, right to left. Additionally, T1-weighted turbo spin-echo (T1-TSE) sequences in the coronal plane were used with parameters: TR, 400 ms; TE, 12 ms; matrix, 512 × 512; FoV, 140 mm; and slice thickness, 1.5 mm. Image analysis was conducted using a Syngo workstation (Siemens Medical Systems) with a high-resolution diagnostic monitor.

### Image analysis

A Syngo workstation (Siemens Medical Systems) with a high-resolution diagnostic monitor was used for MRI scan evaluation. The degree of ossification of the distal radial epiphysis was assessed using the staging criteria described by Vieth et al. [10], with the original methodology’s T2 TSE SPIR sequence replaced by the FSE PD sequence.

**Fig. 1:**
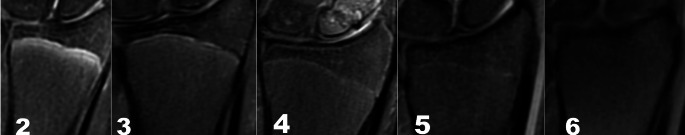
Fast spin-echo proton density (FSE PD)–weighted sequences in coronal orientation on hand wrist MRI: stage 2 to stage 6 for distal radius epiphysis (2–6)

**Fig. 2:**
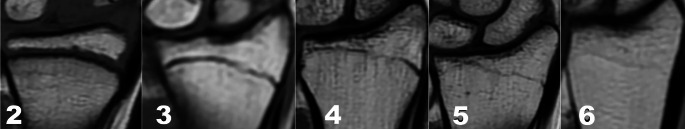
T1-weighted turbo spin echo (T1-TSE) sequences in coronal orientation on hand wrist MRI: stage 2 to stage 6 for distal radius epiphysis (2–6)

- **Stage 2**: Continuous intermediate band in T1-TSE and two continuous or discontinuous hyperintense lines in FSE PD (Figs. 1 and 2).
- **Stage 3**: Discontinuous intermediate band in T1-TSE and two hyperintense, sporadically convening lines in FSE PD (Figs. 1 and 2).
- **Stage 4**: Discontinuous intermediate line in T1-TSE and a thin discontinuous or dotted hyperintense line in FSE PD (Figs. 1 and 2).
- **Stage 5**: Continuous intermediate line in T1-TSE and a discontinuous hyperintense line in FSE PD (Figs. 1 and 2).
- **Stage 6**: Continuous intermediate line in T1-TSE and no signal in FSE PD at the same position (Figs. 1 and 2).

All coronal MRI slices were used for staging, with decisions based on the most developed epiphysis slices.

Two experienced observers, blinded to patient sex and age, evaluated the entire image dataset. The first observer (a radiologist) assessed the dataset twice, while the second observer (a forensic medicine specialist) evaluated it once. Both observers re-evaluated all images after four weeks, remaining blinded to previous staging results.

### Statistical analysis

Statistical analyses were performed using SPSS software (version 17.0; SPSS Inc., Chicago, IL, USA). Descriptive statistics, including minimum age (min), maximum age (max), mean, median, standard deviation (SD), and lower and upper quartiles, were used to describe the phase distribution within the cohort. Spearman’s correlation rank analysis assessed the relationship between age and ossification stage. The Mann–Whitney U test was used to analyze differences between sexes, with *p* < 0.05 considered statistically significant. Intra- and interobserver reliability was measured using Cohen’s kappa (κ) coefficients [20].

## Results

This study evaluated images from 620 patients (286 males and 334 females). The age range was 9.92 to 29.58 years. The mean age of the male patients was 19.27 ± 4.71 years, and that of the female patients was 18.74 ± 4.49 years. Intra- and interobserver agreement for the assessment of the ossification stages were calculated separately. Intraobserver agreement was κ = 0.974, and interobserver reliability was κ = 0.961, indicating very good repeatability and consistency of the method used in this study.

Spearman’s rank correlation analysis showed a significant positive correlation between age and ossification stage for male subjects (Spearman’s correlation coefficient *r* = 0.956, *p* < 0.001), female subjects (Spearman’s correlation coefficient *r* = 0.921, *p* < 0.001), and combined data (Spearman’s correlation coefficient *r* = 0.940, *p* < 0.001).

Statistical analysis of sex differences revealed significant differences for the ages at stage 3 (*p* < 0.01), stage 4 (*p* < 0.01), and stage 5 (*p* < 0.01), but not for stage 2 (*p* = 0.231) and stage 6 (*p* = 0.024).

The age distribution for both sexes is presented in Table 1. Among males, ages ranged from 9 to 29 years, with the highest frequency observed at ages 18 and 19, with 18 and 16 subjects, respectively. For females, ages ranged from 10 to 29 years, with the highest frequency observed at ages 23 and 18, with 15 and 18 subjects, respectively.

**Table 1:** Age distribution of male and female subjects

Age (years)	Male (*N*)	Female (*N*)
9	1	-
10	11	5
11	13	10
12	8	9
13	9	11
14	7	8
15	15	9
16	19	13
17	12	12
18	18	18
19	16	13
20	9	8
21	7	7
22	10	13
23	11	15
24	9	9
25	11	9
26	8	7
27	9	6
28	2	2
29	5	2

The minimum and maximum ages, along with means ± SDs, lower and upper quartiles, and medians at all stages are detailed in Tables 2 and 3 for males and females, respectively.

**Table 2:** Minimum and maximum ages, with means ± SDs, lower and upper quartiles and medians, at all stages for males

Stage	*N*	Mean ± Std Dev	Min	Max	LQ	Median	UQ
2	92	12.89 ± 1.66	9.92	15.67	11.08	13.58	14.50
3	33	16.37 ± 0.91	15.00	18.50	16.00	16.75	17.42
4	72	17.31 ± 1.10	15.00	20.00	16.67	17.92	18.75
5	50	19.60 ± 1.30	17.00	22.75	18.83	19.92	21.00
6	112	24.02 ± 2.39	20.00	29.58	22.33	24.33	26.00

**Table 3:** Minimum and maximum ages, with means ± SDs, lower and upper quartiles and medians, at all stages for females

Stage	*N*	Mean ± Std Dev	Min	Max	LQ	Median	UQ
2	63	12.66 ± 1.53	10.08	15.58	11.00	12.33	13.92
3	26	14.77 ± 1.42	12.33	16.58	13.75	15.00	16.33
4	76	16.76 ± 1.16	14.25	19.92	15.50	16.75	17.92
5	97	19.02 ± 1.40	16.33	22.75	17.67	18.92	20.00
6	119	23.83 ± 2.51	18.42	29.58	22.00	24.00	25.58

## Discussion

This study evaluated the applicability of the Vieth staging system for forensic age estimation using MRI of the distal radial epiphysis in the Turkish population, a methodology previously applied in the German population by Ottow et al. [19]. The findings align with previous research demonstrating the reliability of MRI-based methods in assessing bone age, reinforcing the potential of MRI as a non-invasive, radiation-free, and accurate tool for forensic age estimation [1, 2, 5, 10, 19, 21]. The application of the Vieth staging system in wrist MRI scans is strongly supported by high intra- and inter-observer agreement values (κ = 0.974 and κ = 0.961) for the distal radius epiphysis. Ottow et al. [19] also reported high observer agreement values in their study. However, it should be noted that the current methodologies for age estimation and the specific assessments for age estimation may be influenced by the experience of the observers. It should be taken into account that the observers in this study, as well as those in the study by Ottow et al., had extensive prior experience with multiple MRI methods for age estimation. As Wittschieber et al. [21] previously emphasized, observer experience plays a significant role in the application of forensic age estimation methods. Indeed, as this method is a combination of different MRI weightings and introducing “band” and “line” definitions in its classification, the results may be more affected by observer experience then other methods. The lack of a clear distinction between these two definitions appears to be a limitation of the Vieth method. On the other hand, this study and other studies where the Vieth method was applied to the knee and shoulder also demonstrated high concordance, reducing concerns about the method’s applicability [10, 22–24]. Future studies that classify the number of studies and the experience of practitioners could provide valuable insights into the applicability and reproducibility of the Vieth method.

In the study by Ottow et al. [19], the minimum ages for the distal radial epiphysis were reported as 12.05, 13.69, 15.35, 16.59, and 19.19 years for males and 12.11, 12.30, 14.44, 15.94, and 18.86 years for females. When these results are compared with the findings of the current study, both consistencies and differences are observed. In this study, the minimum ages for males were found to be slightly higher in stages 2, 3, and 4, while the ages for stages 5 and 6 were more closely aligned. For females, the minimum ages in stages 2, 3, and 4 were found to be comparable, but stages 5 and 6 were marginally higher. The comparative minimum age values are presented in Table 4.

**Table 4:** The comparison of the minimum ages and study protocol of the present study with the Ottow et al. [19]

Study	Nationality	Study protocol	Distal radial epiphysis		
			Stage	Male	Female
Ottow et al. [19]	Germany	3.0-T MRI T1-weighted turbo spinecho (T1 TSE) and T2-weighted SPIR sequence in coronal orientation Prospective 695 patients (347 males and 348 females The age range was 12 to 24 years	2	12.05	12.11
			3	13.69	12.30
			4	15.35	14.44
			5	16.59	15.94
			6	19.19	18.86
Present study	Turkey	1.5-T MRI T1-weighted turbo spinecho (T1-TSE) and FSE PD sequence in coronal orientation Retrospective 620 patients (286 males and 334 females) The age range was 9.92 to 29.58 years	2	9.92	10.08
			3	15.00	12.33
			4	15.00	14.25
			5	17.00	16.33
			6	20.00	18.42

The differences in observed minimum ages can be attributed to sample size, population differences, and MR-specific methodological variations. The sample in this study was larger and potentially provided a broader age range and variability. Although this introduces a bias regarding the average values of the stages, as highlighted in the Ottow et al. study, it does not offer an additional advantage for the use of the minimum age concept in forensic age estimation. This is because the minimum age concept is based on identifying the lowest observed age for a specific stage of ossification, and it supports that there are no individuals younger than this in the studied cohort. For readers unfamiliar with the concept, the minimum age is used to determine whether an individual has reached a certain developmental stage, helping to establish forensic age thresholds with certainty.

This study was conducted retrospectively, and it was not possible to obtain socioeconomic or ethnicity data for the study population. Additionally, while the differences between ethnic origins is not considered an effective factor, low socioeconomic status may be associated with delayed ossification [25, 26]. Although the extent to which the study results were affected by socioeconomic status was not directly determined, the delayed ossification ages observed in comparison to the Ottow et al. study might be related to the fact that this study was conducted in a Turkish population with a lower Human Development Index. According to the Human Development Index published by the United Nations in 2023, Turkey is ranked 45th, and Germany is ranked 4th, highlighting a significant difference between the countries where the studies were conducted [27].

Other factors that could influence the study results include the different MRI weightings and magnetic field strengths used. The Vieth method, which relies on hyperintensity and image continuity, might be particularly sensitive to these variations. In this study, a 1.5 Tesla magnetic field was used. The higher magnetic field strength of 3 Tesla MRI scanners might lead to better image quality compared to 1.5 Tesla scanners, potentially offering a higher signal-to-noise ratio and improved spatial resolution. This could enhance the accuracy of ossification stage assessments in forensic age estimation.One of the factors that might influence the differences observed in minimum age values is the MRI sequences used. While Ottow et al. utilized the T2 TSE SPIR sequence, the current study employed the FSE PD sequence. The differences between the T2 TSE SPIR sequence and the FSE PD sequence can play a significant role in age estimation, particularly in terms of image quality and the clarity of bone structures. Indeed, the T2 TSE SPIR sequence, with its fat suppression feature, can provide higher contrast for soft tissues and water-based components, making the epiphyseal-diaphyseal fusion more visible. On the other hand, the FSE PD sequence, which focuses on proton density-weighted imaging, allows for more detailed examination of bone and cartilage structures, potentially offering an advantage in evaluating bone structures for age estimation [28, 29]. These differences suggest that each sequence could yield different results when used for age estimation, with each method potentially offering advantages in specific scenarios.

The results of this study can be compared with previous studies that evaluated the distal radial epiphysis using T1 TSE and FSE PD MRI in a cohort from the Turkish population [18, 30]. Similarly, Timme et al. [31] in a Germany-based study also assessed the distal radius epiphysis using the T1 TSE sequence. They reported minimum age thresholds of 17 years for stages 4 and 4a males and 18 years for stage 4b males using T1-weighted MRI. Ekizoglu et al. [18] found the minimum ages for the distal radius using the FSE PD sequence to be 15–17 years for males and 14–16 years for females. Serin et al. [32], using T1-SE MRI, reported the lowest ages as 15 years for females and 16 years for males. Studies by De Tobel et al. [17], Tomei et al. [14], and Serinelli et al. [15] which used T1-weighted MRI, found significant correlations between chronological age and epiphyseal development but did not specify minimum age thresholds. Among all past studies using T1, T2, and FSE PD sequences for evaluating the distal radius epiphysis, only the staging methodology of Timme et al. [31]., reported a minimum age threshold of 18 years for males at stage 4b. The established minimum ages of 18.2 and 18.6 years require caution due to the low margin. Compared to the results of other methodologies, Ottow et al. [19] reported minimum ages of 18.86 years for females and 19.19 years for males, whereas our current study found minimum ages of 18.42 years for females and 20.00 years for males, providing a safer age margin for determining the age of 18 years at stage 6.

Future research could focus on increasing the sample size and diversity to enhance the generalizability of the findings. Additionally, the development of automated MRI analysis and machine learning methods could reduce observer-dependent errors.

In conclusion, the application of the Vieth staging system to the distal radial epiphysis in the living using MRI can provide critical age estimations for legal and civil proceedings. The high intra- and inter-observer agreement values support the use of this method in forensic applications, contributing to the proven potential of MRI as a non-invasive, radiation-free and accurate tool for age estimation. Future studies should aim to refine these methods and explore their applicability across different populations, ultimately working towards the development of standardized, non-invasive protocols for forensic age estimation.

## Data Availability

The datasets used and/or analyzed during the current study are available from the corresponding author on reasonable request.
